# [3-(4-Chloro­phen­yl)-5-hydr­oxy-5-phenyl-4,5-dihydro-1*H*-pyrazol-1-yl](3-pyrid­yl)methanone

**DOI:** 10.1107/S1600536808037161

**Published:** 2008-11-13

**Authors:** Hoong-Kun Fun, Samuel Robinson Jebas, Jyothi N. Rao, B. Kalluraya

**Affiliations:** a X-ray Crystallography Unit, School of Physics, Universiti Sains Malaysia, 11800 USM, Penang, Malaysia; b Department of Studies in Chemistry, Mangalore University, Mangalagangotri, Mangalore 574 199, India

## Abstract

In the title compound, C_21_H_16_ClN_3_O_2_, the dihedral angles formed by the pyrazole ring with the pyridyl, phenyl­ene and phenyl rings are 6.80 (5), 9.23 (5) and 74.96 (5)°, respectively. The phenyl and phenyl­ene rings are inclined at 80.14 (2)°. Intra­molecular O—H⋯O and C—H⋯N hydrogen bonds generate *S*(6) ring motifs. The crystal packing is strengthened by short inter­molecular O—H⋯N, C—H⋯O hydrogen bonds and π–π stacking inter­actions with centroid–centroid distances of 3.6247 (5)–3.7205 (5) Å, together with inter­molecular short O⋯N contacts [2.7682 (11) Å]. Mol­ecules are linked into infinite chains along [100].

## Related literature

For the biological applications of pyrazoles, see: Kalluraya & Ramesh (2001 ▶); Watanabe *et al.* (1998 ▶); Yuhong & Rajender (2005 ▶). For bond-length data, see: Allen *et al.* (1987 ▶). For graph-set analysis of hydrogen bonding, see: Bernstein *et al.* (1995 ▶).
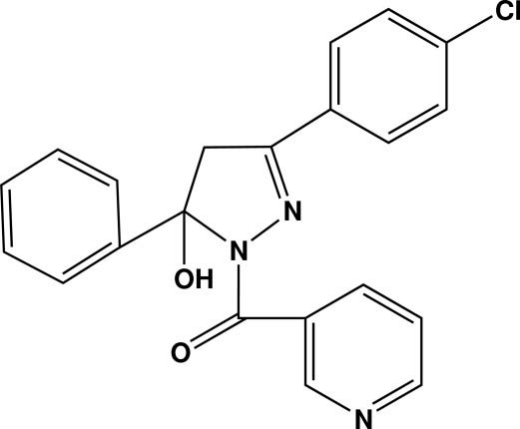


## Experimental

### 

#### Crystal data

C_21_H_16_ClN_3_O_2_
*M* *_r_* = 377.82
Triclinic, 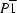

*a* = 7.5916 (1) Å
*b* = 9.7644 (1) Å
*c* = 12.5474 (2) Å
α = 104.424 (1)°
β = 94.960 (1)°
γ = 96.081 (1)°
*V* = 889.55 (2) Å^3^
*Z* = 2
Mo *K*α radiation
μ = 0.24 mm^−1^
*T* = 100.0 (1) K
0.47 × 0.29 × 0.19 mm

#### Data collection

Bruker SMART APEXII CCD area-detector diffractometer
Absorption correction: multi-scan (*SADABS*; Bruker, 2005 ▶) *T*_min_ = 0.896, *T*_max_ = 0.957
20326 measured reflections
6385 independent reflections
5630 reflections with *I* > 2σ(*I*)
*R*_int_ = 0.023

#### Refinement

*R*[*F* ^2^ > 2σ(*F* ^2^)] = 0.037
*wR*(*F* ^2^) = 0.103
*S* = 1.04
6385 reflections
248 parameters
1 restraint
H atoms treated by a mixture of independent and constrained refinement
Δρ_max_ = 0.54 e Å^−3^
Δρ_min_ = −0.26 e Å^−3^

### 

Data collection: *APEX2* (Bruker, 2005 ▶); cell refinement: *APEX2*; data reduction: *SAINT* (Bruker, 2005 ▶); program(s) used to solve structure: *SHELXTL* (Sheldrick, 2008 ▶); program(s) used to refine structure: *SHELXTL*; molecular graphics: *SHELXTL*; software used to prepare material for publication: *SHELXTL* and *PLATON* (Spek, 2003 ▶).

## Supplementary Material

Crystal structure: contains datablocks global, I. DOI: 10.1107/S1600536808037161/ng2515sup1.cif

Structure factors: contains datablocks I. DOI: 10.1107/S1600536808037161/ng2515Isup2.hkl

Additional supplementary materials:  crystallographic information; 3D view; checkCIF report

## Figures and Tables

**Table 1:** Hydrogen-bond geometry (Å, °)

*D*—H⋯*A*	*D*—H	H⋯*A*	*D*⋯*A*	*D*—H⋯*A*
O2—H1*O*2⋯O1	0.824 (13)	2.340 (14)	2.8463 (9)	120.3 (13)
O2—H1*O*2⋯N3^i^	0.824 (13)	2.027 (14)	2.7682 (11)	149.5 (14)
C8—H8*A*⋯O2^ii^	0.97	2.55	3.4836 (11)	163
C13—H13*A*⋯O1^iii^	0.93	2.46	3.3294 (12)	156
C21—H21*A*⋯N1	0.93	2.21	2.8600 (12)	127
C14—H14*A*⋯*Cg*2^iv^	0.93	2.90	3.6968 (11)	144

Symmetry codes: (i) 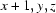; (ii) 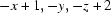; (iii) 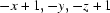; (iv) 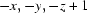. *Cg*2 is the centroid of the N3/C17–C21 ring.

**Fig. 1.**
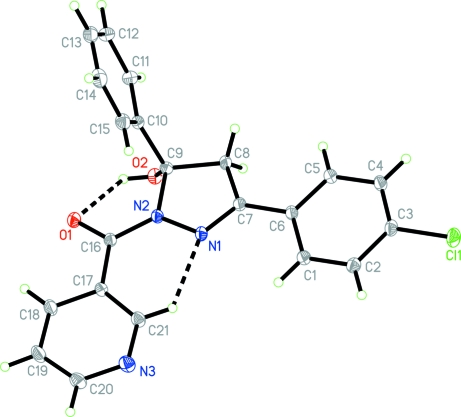
The molecular structure of the title compound, showing 50% probability displacement ellipsoids and the atom numbering scheme.

**Fig. 2.**
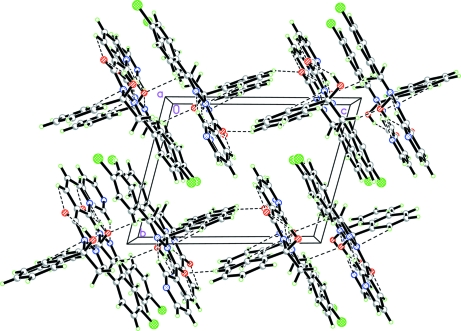
The crystal packing of the title compound,viewed down the a axis.

## supplementary crystallographic information

### Comment

Heterocyclic compounds occur very widely in nature and are essential to life. Nitrogen-containing heterocyclic molecules constitute the largest portion of chemical entities, which are part of many natural products, fine chemicals, and biologically active pharmaceuticals vital for enhancing the quality of life. 4,5-Dihydro-pyrazoles, pyrazolidines and 1,2-dihydro-phthalazines are important classes of heterocycles useful as pesticides, anticonvulsants, and potent vasorelaxing agents (Kalluraya *et al.*, 2001; Watanabe *et al.*, 1998; Yuhong & Rajender, 2005). The pyrazoline function is quite stable and has inspired chemists to utilize this stable fragment in bioactive moieties to synthesize new compounds. Prompted by these review, we have synthesized this new substituted pyrazoline derivative and report its crystal structure.

Bond lengths and angles in (I) (Fig. 1) are found to have normal values (Allen *et al.*, 1987). The dihedral angle formed by the pyrazole (N1/N2/C7—C9) ring with the pyridine ring (N3/C17—C21) and the two benzene rings (C1—C6; C10—C15) are 6.80 (5), 9.23 (5) and 74.96 (5)° respectively. The benzene rings (C1—C6; C10—C15) form dihedral angle of 80.14 (2)°, indicating that they are inclined to each other. Intramolecular C—H···N and O—H···O hydrogen bonds generate S(6) ring motifs. (Bernstein *et al.*, 1995).

The crystal packing is consolidated by intermolecular O—H···N and C—H···O hydrogen bonding (Table 1). Furthermore, the packing is strengthened by π—π stacking interactions involving the pyrazole (N1—N2/C7—C9) (*Cg*1) ring and the symmetry related benzene (C10—C15) (*Cg*4) ring [*Cg*1···*Cg*4^v^=3.7787 (6) Å; symmetry code: (v) *X*,Y,*Z*]; pyridine (N3/C17—C21) (*Cg*2) ring and the symmetry related benzene (C1—C6) (*Cg*3) ring [*Cg*2···*Cg*3^vi^=3.6247 (5) Å; symmetry code: (vi) –*X*,-Y,2-*Z*] and between symmetry related benzene (C1—C6) (*Cg*3) rings [*Cg*3···*Cg*3^vii^ = 3.7205 (5) Å; symmetry code: (vii) –*X*,1-Y,2-*Z*] together with intermolecular O···N = 2.7682 (11)Å short contacts. In the crystal packing, the molecules are linked into infinite one dimensional chains along the [100] direction (Fig 2).

### Experimental

A mixture of 1-phenyl-3-(4-chloro phenyl)-2,3-di-bromo propan-1-one (0.01 mol), nicotinic hydrazide (0.01 mol) and trimethylamine (0.04 mol) in ethanol (30 mL) was refluxed for 8 h. The contents were poured onto crushed ice with stirring. The solid mass separated was collected and recrystallized from ethanol.

### Refinement

The hydroxy H atoms were located in a difference map and refined with restraints of O—H=0.82 (1) Å. The remaining H atoms were positioned geometrically [C—H=0.93Å (aromatic) or 0.97Å (methylene)] and refined using a riding model, with *U*_iso_(H)=1.2U_equ_(aromatic C, methylene).

### Crystal data

**Table:**

C_21_H_16_ClN_3_O_2_	*Z* = 2
*M**_r_* = 377.82	*F*_000_ = 392
Triclinic, *P*1	*D*_x_ = 1.411 Mg m^−^^3^
Hall symbol: -P 1	Mo *K*α radiation λ = 0.71073 Å
*a* = 7.5916 (1) Å	Cell parameters from 9943 reflections
*b* = 9.7644 (1) Å	θ = 3.1–37.5º
*c* = 12.5474 (2) Å	µ = 0.24 mm^−^^1^
α = 104.424 (1)º	*T* = 100.0 (1) K
β = 94.960 (1)º	Block, colourless
γ = 96.081 (1)º	0.47 × 0.29 × 0.19 mm
*V* = 889.55 (2) Å^3^	

### Data collection

**Table:**

Bruker SMART APEXII CCD area-detector diffractometer	6385 independent reflections
Radiation source: fine-focus sealed tube	5630 reflections with *I* > 2σ(*I*)
Monochromator: graphite	*R*_int_ = 0.023
*T* = 100.0(1) K	θ_max_ = 32.5º
φ and ω scans	θ_min_ = 1.7º
Absorption correction: multi-scan(SADABS; Bruker, 2005)	*h* = −11→11
*T*_min_ = 0.896, *T*_max_ = 0.957	*k* = −14→14
20326 measured reflections	*l* = −18→18

### Refinement

**Table:**

Refinement on *F*^2^	Secondary atom site location: difference Fourier map
Least-squares matrix: full	Hydrogen site location: inferred from neighbouring sites
*R*[*F*^2^ > 2σ(*F*^2^)] = 0.037	H atoms treated by a mixture of independent and constrained refinement
*wR*(*F*^2^) = 0.104	*w* = 1/[σ^2^(*F*_o_^2^) + (0.0577*P*)^2^ + 0.2362*P*] where *P* = (*F*_o_^2^ + 2*F*_c_^2^)/3
*S* = 1.04	(Δ/σ)_max_ < 0.001
6385 reflections	Δρ_max_ = 0.54 e Å^−^^3^
248 parameters	Δρ_min_ = −0.26 e Å^−^^3^
1 restraint	Extinction correction: none
Primary atom site location: structure-invariant direct methods	

### Special details

**Table:**

Experimental. The data was collected with the Oxford Cyrosystem Cobra low-temperature attachment.
Geometry. All e.s.d.'s (except the e.s.d. in the dihedral angle between two l.s. planes) are estimated using the full covariance matrix. The cell e.s.d.'s are taken into account individually in the estimation of e.s.d.'s in distances, angles and torsion angles; correlations between e.s.d.'s in cell parameters are only used when they are defined by crystal symmetry. An approximate (isotropic) treatment of cell e.s.d.'s is used for estimating e.s.d.'s involving l.s. planes.
Refinement. Refinement of *F*^2^ against ALL reflections. The weighted *R*-factor *wR* and goodness of fit *S* are based on *F*^2^, conventional *R*-factors *R* are based on *F*, with *F* set to zero for negative *F*^2^. The threshold expression of *F*^2^ > σ(*F*^2^) is used only for calculating *R*-factors(gt) *etc*. and is not relevant to the choice of reflections for refinement. *R*-factors based on *F*^2^ are statistically about twice as large as those based on *F*, and *R*- factors based on ALL data will be even larger.

### Fractional atomic coordinates and isotropic or equivalent isotropic displacement parameters (Å^2^)

**Table:**

	*x*	*y*	*z*	*U*_iso_*/*U*_eq_	
Cl1	−0.18759 (3)	0.58111 (2)	1.24148 (2)	0.02272 (7)	
O1	0.24543 (9)	−0.22304 (7)	0.66899 (5)	0.01553 (13)	
O2	0.47798 (8)	−0.05281 (7)	0.85593 (5)	0.01450 (12)	
N1	0.05961 (10)	0.04156 (8)	0.85564 (6)	0.01321 (13)	
N2	0.17594 (9)	−0.03622 (8)	0.79391 (6)	0.01266 (13)	
N3	−0.36717 (10)	−0.27300 (9)	0.73008 (7)	0.01823 (15)	
C1	−0.11452 (12)	0.23011 (9)	1.01516 (7)	0.01448 (15)	
H1A	−0.1821	0.1448	0.9752	0.017*	
C2	−0.19255 (12)	0.32951 (10)	1.08935 (7)	0.01548 (16)	
H2A	−0.3119	0.3113	1.0994	0.019*	
C3	−0.08985 (13)	0.45689 (9)	1.14851 (7)	0.01577 (16)	
C4	0.08865 (13)	0.48666 (9)	1.13548 (7)	0.01624 (16)	
H4A	0.1555	0.5722	1.1756	0.019*	
C5	0.16553 (12)	0.38617 (9)	1.06134 (7)	0.01500 (15)	
H5A	0.2852	0.4047	1.0523	0.018*	
C6	0.06584 (11)	0.25743 (9)	1.00002 (7)	0.01290 (14)	
C7	0.15147 (11)	0.15330 (9)	0.92314 (7)	0.01247 (14)	
C8	0.34900 (11)	0.16151 (9)	0.91685 (7)	0.01392 (15)	
H8A	0.4118	0.1521	0.9848	0.017*	
H8B	0.3961	0.2510	0.9030	0.017*	
C9	0.36523 (11)	0.03425 (9)	0.81857 (7)	0.01211 (14)	
C10	0.42505 (12)	0.08305 (9)	0.71939 (7)	0.01367 (15)	
C11	0.60717 (13)	0.11624 (10)	0.71445 (8)	0.01826 (17)	
H11A	0.6901	0.1036	0.7690	0.022*	
C12	0.66489 (15)	0.16839 (11)	0.62776 (9)	0.0236 (2)	
H12A	0.7863	0.1903	0.6248	0.028*	
C13	0.54216 (16)	0.18775 (11)	0.54588 (8)	0.0245 (2)	
H13A	0.5810	0.2208	0.4874	0.029*	
C14	0.36126 (16)	0.15747 (11)	0.55197 (8)	0.0227 (2)	
H14A	0.2786	0.1718	0.4979	0.027*	
C15	0.30233 (13)	0.10574 (10)	0.63848 (8)	0.01771 (16)	
H15A	0.1807	0.0863	0.6422	0.021*	
C16	0.12920 (11)	−0.16587 (9)	0.71942 (7)	0.01213 (14)	
C17	−0.05871 (11)	−0.24069 (9)	0.69814 (7)	0.01225 (14)	
C18	−0.08787 (12)	−0.36918 (10)	0.61570 (8)	0.01686 (16)	
H18A	0.0050	−0.4016	0.5768	0.020*	
C19	−0.25619 (13)	−0.44831 (10)	0.59202 (8)	0.01974 (18)	
H19A	−0.2776	−0.5344	0.5376	0.024*	
C20	−0.39171 (12)	−0.39616 (10)	0.65129 (8)	0.01884 (17)	
H20A	−0.5044	−0.4492	0.6355	0.023*	
C21	−0.20374 (11)	−0.19778 (10)	0.75323 (8)	0.01554 (16)	
H21A	−0.1862	−0.1128	0.8088	0.019*	
H1O2	0.488 (2)	−0.1208 (13)	0.8037 (10)	0.026 (4)*	

### Atomic displacement parameters (Å^2^)

**Table:**

	*U*^11^	*U*^22^	*U*^33^	*U*^12^	*U*^13^	*U*^23^
Cl1	0.02952 (12)	0.01698 (11)	0.02199 (12)	0.00842 (8)	0.01020 (9)	0.00106 (8)
O1	0.0147 (3)	0.0150 (3)	0.0160 (3)	0.0030 (2)	0.0043 (2)	0.0011 (2)
O2	0.0147 (3)	0.0148 (3)	0.0141 (3)	0.0036 (2)	0.0005 (2)	0.0036 (2)
N1	0.0140 (3)	0.0118 (3)	0.0129 (3)	0.0026 (2)	0.0031 (2)	0.0008 (2)
N2	0.0110 (3)	0.0120 (3)	0.0133 (3)	0.0011 (2)	0.0025 (2)	0.0000 (2)
N3	0.0120 (3)	0.0183 (4)	0.0217 (4)	0.0009 (3)	−0.0001 (3)	0.0015 (3)
C1	0.0157 (3)	0.0130 (4)	0.0143 (4)	0.0018 (3)	0.0019 (3)	0.0028 (3)
C2	0.0169 (4)	0.0154 (4)	0.0151 (4)	0.0038 (3)	0.0035 (3)	0.0045 (3)
C3	0.0218 (4)	0.0131 (4)	0.0134 (4)	0.0057 (3)	0.0045 (3)	0.0030 (3)
C4	0.0214 (4)	0.0118 (4)	0.0146 (4)	0.0016 (3)	0.0029 (3)	0.0015 (3)
C5	0.0170 (4)	0.0126 (4)	0.0142 (4)	0.0007 (3)	0.0022 (3)	0.0018 (3)
C6	0.0156 (3)	0.0113 (3)	0.0115 (3)	0.0022 (3)	0.0019 (3)	0.0023 (3)
C7	0.0141 (3)	0.0115 (3)	0.0118 (3)	0.0018 (3)	0.0019 (3)	0.0027 (3)
C8	0.0135 (3)	0.0138 (4)	0.0123 (3)	0.0003 (3)	0.0017 (3)	0.0000 (3)
C9	0.0111 (3)	0.0126 (3)	0.0121 (3)	0.0006 (3)	0.0012 (3)	0.0026 (3)
C10	0.0172 (4)	0.0110 (3)	0.0124 (3)	0.0010 (3)	0.0033 (3)	0.0021 (3)
C11	0.0186 (4)	0.0180 (4)	0.0175 (4)	−0.0013 (3)	0.0045 (3)	0.0039 (3)
C12	0.0289 (5)	0.0178 (4)	0.0233 (5)	−0.0033 (4)	0.0120 (4)	0.0035 (3)
C13	0.0433 (6)	0.0135 (4)	0.0177 (4)	−0.0002 (4)	0.0113 (4)	0.0047 (3)
C14	0.0382 (6)	0.0153 (4)	0.0153 (4)	0.0045 (4)	0.0017 (4)	0.0055 (3)
C15	0.0228 (4)	0.0149 (4)	0.0156 (4)	0.0031 (3)	0.0017 (3)	0.0043 (3)
C16	0.0133 (3)	0.0116 (3)	0.0111 (3)	0.0014 (3)	0.0011 (3)	0.0023 (3)
C17	0.0122 (3)	0.0115 (3)	0.0123 (3)	0.0017 (3)	0.0002 (3)	0.0022 (3)
C18	0.0165 (4)	0.0141 (4)	0.0169 (4)	0.0008 (3)	0.0013 (3)	−0.0009 (3)
C19	0.0192 (4)	0.0150 (4)	0.0203 (4)	−0.0015 (3)	−0.0008 (3)	−0.0017 (3)
C20	0.0149 (4)	0.0181 (4)	0.0205 (4)	−0.0017 (3)	−0.0023 (3)	0.0025 (3)
C21	0.0126 (3)	0.0143 (4)	0.0177 (4)	0.0016 (3)	0.0006 (3)	0.0007 (3)

### Geometric parameters (Å, °)

**Table:**

Cl1—C3	1.7373 (9)	C8—H8A	0.9700
O1—C16	1.2311 (10)	C8—H8B	0.9700
O2—C9	1.3994 (11)	C9—C10	1.5262 (12)
O2—H1O2	0.823 (9)	C10—C15	1.3928 (13)
N1—C7	1.2924 (11)	C10—C11	1.3965 (12)
N1—N2	1.3870 (10)	C11—C12	1.3948 (13)
N2—C16	1.3638 (11)	C11—H11A	0.9300
N2—C9	1.4972 (11)	C12—C13	1.3885 (17)
N3—C20	1.3367 (12)	C12—H12A	0.9300
N3—C21	1.3422 (11)	C13—C14	1.3866 (16)
C1—C2	1.3880 (12)	C13—H13A	0.9300
C1—C6	1.4053 (12)	C14—C15	1.3938 (13)
C1—H1A	0.9300	C14—H14A	0.9300
C2—C3	1.3929 (13)	C15—H15A	0.9300
C2—H2A	0.9300	C16—C17	1.5021 (11)
C3—C4	1.3879 (13)	C17—C18	1.3957 (12)
C4—C5	1.3889 (12)	C17—C21	1.3966 (12)
C4—H4A	0.9300	C18—C19	1.3889 (12)
C5—C6	1.4003 (12)	C18—H18A	0.9300
C5—H5A	0.9300	C19—C20	1.3876 (14)
C6—C7	1.4652 (12)	C19—H19A	0.9300
C7—C8	1.5032 (12)	C20—H20A	0.9300
C8—C9	1.5412 (12)	C21—H21A	0.9300
			
C9—O2—H1O2	108.8 (10)	C10—C9—C8	111.83 (7)
C7—N1—N2	108.27 (7)	C15—C10—C11	119.31 (8)
C16—N2—N1	125.12 (7)	C15—C10—C9	121.51 (8)
C16—N2—C9	121.54 (7)	C11—C10—C9	118.99 (8)
N1—N2—C9	113.32 (7)	C12—C11—C10	120.09 (9)
C20—N3—C21	118.29 (8)	C12—C11—H11A	120.0
C2—C1—C6	120.46 (8)	C10—C11—H11A	120.0
C2—C1—H1A	119.8	C13—C12—C11	120.40 (10)
C6—C1—H1A	119.8	C13—C12—H12A	119.8
C1—C2—C3	119.15 (8)	C11—C12—H12A	119.8
C1—C2—H2A	120.4	C14—C13—C12	119.51 (9)
C3—C2—H2A	120.4	C14—C13—H13A	120.2
C4—C3—C2	121.67 (8)	C12—C13—H13A	120.2
C4—C3—Cl1	119.23 (7)	C13—C14—C15	120.49 (10)
C2—C3—Cl1	119.10 (7)	C13—C14—H14A	119.8
C3—C4—C5	118.69 (8)	C15—C14—H14A	119.8
C3—C4—H4A	120.7	C10—C15—C14	120.18 (9)
C5—C4—H4A	120.7	C10—C15—H15A	119.9
C4—C5—C6	121.12 (8)	C14—C15—H15A	119.9
C4—C5—H5A	119.4	O1—C16—N2	118.64 (8)
C6—C5—H5A	119.4	O1—C16—C17	119.41 (8)
C5—C6—C1	118.91 (8)	N2—C16—C17	121.95 (7)
C5—C6—C7	119.70 (8)	C18—C17—C21	117.32 (8)
C1—C6—C7	121.37 (8)	C18—C17—C16	115.35 (7)
N1—C7—C6	121.37 (8)	C21—C17—C16	127.30 (8)
N1—C7—C8	113.88 (7)	C19—C18—C17	119.68 (9)
C6—C7—C8	124.71 (7)	C19—C18—H18A	120.2
C7—C8—C9	103.47 (7)	C17—C18—H18A	120.2
C7—C8—H8A	111.1	C20—C19—C18	118.56 (9)
C9—C8—H8A	111.1	C20—C19—H19A	120.7
C7—C8—H8B	111.1	C18—C19—H19A	120.7
C9—C8—H8B	111.1	N3—C20—C19	122.80 (8)
H8A—C8—H8B	109.0	N3—C20—H20A	118.6
O2—C9—N2	111.30 (7)	C19—C20—H20A	118.6
O2—C9—C10	113.38 (7)	N3—C21—C17	123.34 (8)
N2—C9—C10	110.73 (7)	N3—C21—H21A	118.3
O2—C9—C8	108.31 (7)	C17—C21—H21A	118.3
N2—C9—C8	100.55 (6)		
			
C7—N1—N2—C16	175.28 (8)	N2—C9—C10—C15	21.78 (11)
C7—N1—N2—C9	−3.15 (10)	C8—C9—C10—C15	−89.47 (10)
C6—C1—C2—C3	0.11 (13)	O2—C9—C10—C11	−37.34 (11)
C1—C2—C3—C4	−0.26 (14)	N2—C9—C10—C11	−163.27 (8)
C1—C2—C3—Cl1	−179.91 (7)	C8—C9—C10—C11	85.48 (10)
C2—C3—C4—C5	0.00 (14)	C15—C10—C11—C12	−1.49 (14)
Cl1—C3—C4—C5	179.66 (7)	C9—C10—C11—C12	−176.55 (8)
C3—C4—C5—C6	0.41 (14)	C10—C11—C12—C13	0.03 (15)
C4—C5—C6—C1	−0.56 (13)	C11—C12—C13—C14	1.25 (15)
C4—C5—C6—C7	−179.54 (8)	C12—C13—C14—C15	−1.05 (15)
C2—C1—C6—C5	0.29 (13)	C11—C10—C15—C14	1.69 (14)
C2—C1—C6—C7	179.26 (8)	C9—C10—C15—C14	176.63 (8)
N2—N1—C7—C6	179.99 (7)	C13—C14—C15—C10	−0.43 (14)
N2—N1—C7—C8	−1.91 (10)	N1—N2—C16—O1	179.18 (8)
C5—C6—C7—N1	−171.57 (8)	C9—N2—C16—O1	−2.51 (12)
C1—C6—C7—N1	9.47 (13)	N1—N2—C16—C17	−1.32 (13)
C5—C6—C7—C8	10.55 (13)	C9—N2—C16—C17	176.99 (7)
C1—C6—C7—C8	−168.42 (8)	O1—C16—C17—C18	−3.55 (12)
N1—C7—C8—C9	5.84 (10)	N2—C16—C17—C18	176.95 (8)
C6—C7—C8—C9	−176.14 (8)	O1—C16—C17—C21	174.65 (9)
C16—N2—C9—O2	−57.55 (10)	N2—C16—C17—C21	−4.85 (14)
N1—N2—C9—O2	120.95 (8)	C21—C17—C18—C19	−0.15 (14)
C16—N2—C9—C10	69.54 (10)	C16—C17—C18—C19	178.25 (8)
N1—N2—C9—C10	−111.97 (8)	C17—C18—C19—C20	0.35 (15)
C16—N2—C9—C8	−172.11 (8)	C21—N3—C20—C19	−0.70 (15)
N1—N2—C9—C8	6.39 (9)	C18—C19—C20—N3	0.08 (16)
C7—C8—C9—O2	−123.48 (7)	C20—N3—C21—C17	0.92 (14)
C7—C8—C9—N2	−6.68 (8)	C18—C17—C21—N3	−0.51 (14)
C7—C8—C9—C10	110.87 (8)	C16—C17—C21—N3	−178.68 (9)
O2—C9—C10—C15	147.71 (8)		

### Hydrogen-bond geometry (Å, °)

**Table:**

*D*—H···*A*	*D*—H	H···*A*	*D*···*A*	*D*—H···*A*
O2—H1O2···O1	0.824 (13)	2.340 (14)	2.8463 (9)	120.3 (13)
O2—H1O2···N3^i^	0.824 (13)	2.027 (14)	2.7682 (11)	149.5 (14)
C8—H8A···O2^ii^	0.97	2.55	3.4836 (11)	163
C13—H13A···O1^iii^	0.93	2.46	3.3294 (12)	156
C21—H21A···N1	0.93	2.21	2.8600 (12)	127
C14—H14A···Cg2^iv^	0.93	2.90	3.6968 (11)	144

Symmetry codes: (i) *x*+1, *y*, *z*; (ii) −*x*+1, −*y*, −*z*+2; (iii) −*x*+1, −*y*, −*z*+1; (iv) −*x*, −*y*, −*z*+1.
